# Use and outcome of TIPS in hospitalized patients in Germany: A Nationwide study (2007–2018)

**DOI:** 10.1097/HC9.0000000000000237

**Published:** 2023-09-15

**Authors:** Wenyi Gu, Yasmin Zeleke, Hannah Hortlik, Louisa Schaaf, Frank E. Uschner, Martin Schulz, Michael Tischendorf, Kai-Henrik Peiffer, Maximilian Joseph Brol, Markus Kimmann, Thomas Vogl, Michael Köhler, Carsten Meyer, Alexander Gerbes, Martin Rössle, Wim Laleman, Alexander Zipprich, Christian Steib, Michael Praktiknjo, Jonel Trebicka

**Affiliations:** 1 Department of Internal Medicine B, University Hospital Muenster, Muenster, Germany; 2 Department of Internal Medicine I, University Hospital Frankfurt, Goethe University Frankfurt, Frankfurt am Main, Germany; 3 Institute of Diagnostic and Interventional Radiology, University Hospital Frankfurt, Frankfurt am Main, Germany; 4 Clinic for Radiology, University Hospital Muenster, Muenster, Germany; 5 Clinic for Radiology, University Hospital Bonn, Bonn, Germany; 6 Department of Medicine II, University Clinic Munich LMU, Munich, Germany; 7 Department of Internal Medicine II, Faculty of Medicine, Medical Centre University of Freiburg, University of Freiburg, Freiburg, Germany; 8 Department of Gastroenterology and Hepatology, University Hospitals Leuven, KU Leuven, Leuven, Belgium; 9 Department of Internal Medicine IV (Gastroenterology, Hepatology and Infectious Diseases), Jena University Hospital, Friedrich-Schiller-University Jena, Jena, Germany; 10 European Foundation for Study of Chronic Liver Failure, Barcelona, Spain; 11 Department of Gastroenterology and Hepatology, Odense University Hospital, Odense, Denmark

## Abstract

**Background:**

The number of complications in patients admitted for cirrhosis has increased over time. Portal hypertension is the driver of many complications of cirrhosis. TIPS placement is the most effective treatment of portal hypertension. The aim of this study was to analyze the use and impact of TIPS placement in the last decade in a nationwide study in Germany.

**Methods:**

We analyzed 14,598 admissions of patients for TIPS insertions in Germany from 2007 to 2018 using the DRG system, 12,877 out of 2,000,765 total admissions of patients with cirrhosis. All diagnoses and procedures were coded according to ICD-10-CM and OPS codes. The data were analyzed, focusing on the number of admissions and in-hospital mortality.

**Results:**

The number of TIPS placements increased over the last decade. In-hospital mortality of cirrhotic patients with TIPS decreased when it was placed for severe bleeding (15.2% [TIPS] vs. 19.5% [endoscopy treatment]), ascites (8.7% [TIPS] vs. 14.4% [paracentesis]), and hepatorenal syndrome (HRS) (17.1% [TIPS] vs. 43.3% [no-TIPS]). In the case of bleeding, TIPS significantly decreased in-hospital mortality and also in ascites and HRS. During hospitalization, 22.6% admissions of patients with TIPS insertion showed HE. However, in-hospital mortality in patients admitted with HE grades 1 or 2 and TIPS was lower than in patients without TIPS. In the logistic regression, a higher HE grade(3 and 4), infection, and circulatory disease were found to be independently associated with in-hospital mortality in patients with TIPS insertion.

**Conclusion:**

Our nationwide study demonstrates that TIPS insertion is increasingly used in Germany. TIPS improves outcomes, especially in patients with ascites and HRS, regardless of lower HE grades, while higher HE grades, infection, and circulatory diseases seem to be associated with risk of in-hospital mortality.

## Graphical abstract

**Figure:**
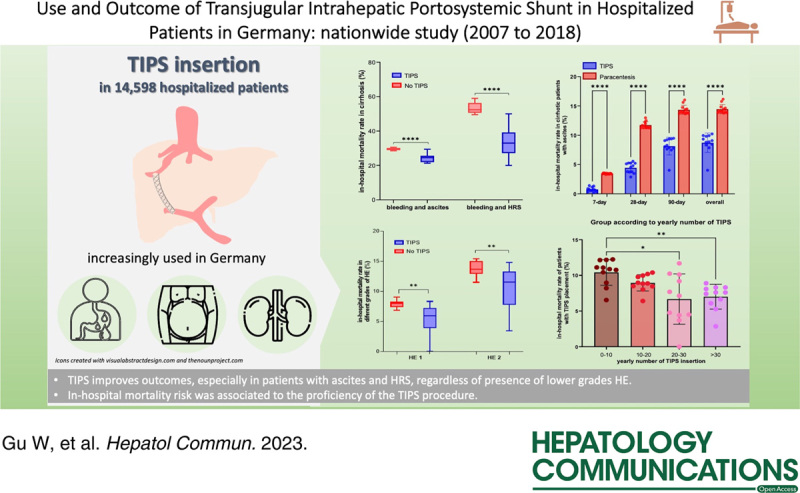

## INTRODUCTION

Portal hypertension (PHT) is the consequence of either advanced chronic liver diseases or non-cirrhotic causes, such as vascular disorders of the liver,^1–3^ and is associated with high morbidity and mortality.^4–6^

The development of bleeding from gastroesophageal varices is a major complication of PHT.^7,8^ However, the frequency of variceal bleeding has decreased over time, at least in Germany. By contrast, other complications of PHT, such as ascites, hepatorenal syndrome (HRS), and PVT, have increased in the last decade.^3,6,9,10^

The insertion of TIPS is the most effective treatment for PHT.^11^ Studies have shown that TIPS insertion increases the survival of patients with recurrent ascites, also in selected patients with refractory ascites and variceal bleeding.^12–16^

However, the occurrence of overt HE is a contraindication for TIPS as it may aggravate post-shunt encephalopathy, which has been reported to be frequent in patients after TIPS, with an incidence ranging from 15% to 48%.^17^ The application of TIPS for different indications has not been studied to date.^18–22^ Although TIPS implantation has been consolidated in clinical practice guidelines recommendations, nationwide data outside of clinical trials on TIPS application and outcomes of patients are scarce.^3,6,11,23^

This nationwide study aims to investigate and report on changes and trends of TIPS implantation in Germany from 2007 (the start of the diagnosis-related group [DRG] record for TIPS) to 2018 and to study the impact of HE and other comorbidities on in-hospital mortality of patients with TIPS placement.

## METHODS

### Study design and data source

This nationwide observational study analyzes the hospitalization of the German population from 2007 to 2018. In 2019, permission for access to the data used in this study was agreed upon between the University of Frankfurt, Germany and the Federal Statistics Office of Wiesbaden, Germany. The data were collected using operational DRG and procedure key codes (OPS). We analyzed sociodemographic background and clinical information, such as main and secondary diagnoses, procedures, and the reason for hospital discharge, that is, actual discharge or death of the patient.

The International Classification of Diseases, tenth edition, Clinical Modification (ICD-10-CM) codes were used in the coding of all data.

### Study population

All admissions in the designated time era in Germany were considered part of the study population. Further assessment focused on admissions of patients with cirrhosis who either did or did not receive a TIPS, while liver cirrhosis was defined as ICD code K74 or K70.3 in the main or secondary diagnosis. TIPS was defined as OPS codes 8-839.81, 8-839.82, 8-839.83, 8-839.85, 8-839.87, 8-839.88, 8-839.89, 8-839.8a, 8-839.8x and 8-839.8. TIPS placement was further separated into 2 groups, covered TIPS insertions and uncovered TIPS insertions, which have been coded since 2012. For different indications of TIPS, bleeding patients with endoscopic treatment and patients with paracentesis in ascites were used as a control group. In addition, other disease conditions with their respective ICD codes (infections, cardiovascular disease, other heart diseases, respiratory diseases, HE, hypalbuminemia, and hyperbilirubinemia) were used to highlight the impact of TIPS on the primary outcome, that is, all-cause hospital mortality. The study admissions were tracked during the entire hospitalization period until discharge or in-hospital death. Any additional diseases were coded according to the ICD-10 DRG coding system and are displayed in Supplemental Table S1, http://links.lww.com/HC9/A491.

### Statistical analysis

Hospital admissions were analyzed with variables presented in either total numbers or percentages. The analysis-based admissions of patients coded with TIPS insertion can be considered as individual patient-based since the TIPS procedure was not coded repeatedly. To compare categorical parameters, chi-square tests were used. The overall mean mortality rate from different years was compared using the nonparametric Mann-Whitney *U* test with multiple tests adjustment for *p* value. Furthermore, we used simple linear regression to analyze correlations between variables and/or mortality changes and development during the observation period. The age-standardized mortality rate was calculated based on the age-specific (mortality) rates for each age group by dividing the number of deaths by the German population of the respective year. The impact of TIPS on in-hospital mortality and other diseases was tested through multivariable logistic regression. For reference, *p* < 0.05 was defined as statistically significant. All statistical analyses were performed with SAS software (version 3.8; SAS Institute Inc., Cary, NC).

## RESULTS

### Demographic characteristics and indications of patients admitted for TIPS insertion

**FIGURE 1:**
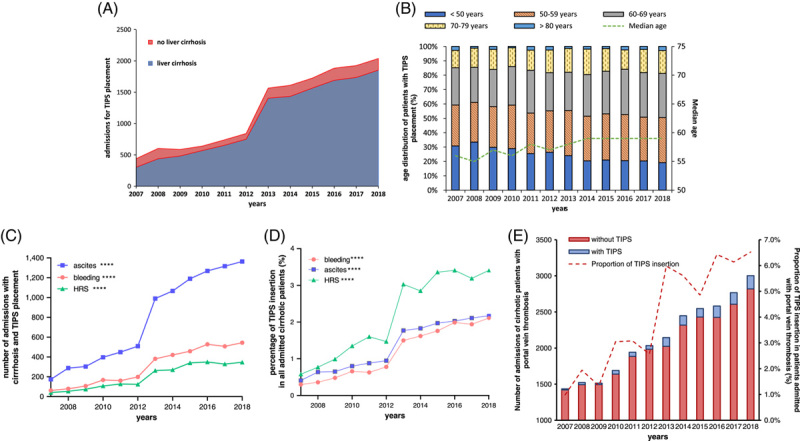
(A) Numbers of admissions of TIPS insertion in patients with and without liver cirrhosis from 2007 to 2018. (B) The proportion of admission of patients for TIPS insertion in each age group from 2007 to 2018. (C) The number of admissions of cirrhotic patients for TIPS insertion with different indications. (D) The proportion of admissions of cirrhotic patients for TIPS insertion with different indications in admissions of patients with ascites, bleeding, or HRS. (E) Number of admissions of patients with PVT with or without TIPS insertion and proportion of TIPS insertions among all admissions of non-HCC patients with PVT. ****p < 0.0001 with linear regression. Abbreviation: HRS, hepatorenal syndrome.

Between 2007 and 2018, 14,598 patients received TIPS insertions, the vast majority of whom (88.7% [12,877]) had liver cirrhosis (Table 1). The yearly number of admissions was almost 5 times higher in 2018 (2037) than in 2007 (439) and increased significantly during the observation period, mainly due to patients with liver cirrhosis (*p* < 0.0001) (Figure 1A), whereas the number of admissions of non-cirrhotic patients who received TIPS increased by 35.3%, that is, not significantly, during the observation period (from 136 to 184) (Figure 1A).

**TABLE 1:** Demographic data and number of patients with TIPS insertion, number of different indications for TIPS insertion, and complications or comorbidities of TIPS from 2007 to 2018

	2007	2008	2009	2010	2011	2012	2013	2014	2015	2016	2017	2018	*P*
TIPS insertion, n	1096	1209	1243	1283	1477	1648	1624	1672	1805	1964	2003	2093	
Median age, year	56	55	57	56	58	57	58	59	59	59	59	59	
Male percentage, %	68.98	68.49	68.88	67.14	69.48	64.58	67.90	65.06	67.03	65.80	67.95	66.81	
Percentage of liver cirrhosis, %	69.02	72.52	82.11	89.05	88.23	89.19	89.78	89.01	90.72	89.70	90.08	90.97	
Indications													
Bleeding	20.46	18.04	22.20	29.53	24.69	26.50	27.19	29.36	29.27	31.24	29.28	29.36	
Ascites	57.43	65.75	63.07	69.77	68.87	67.78	70.46	74.34	76.04	75.09	75.91	73.61	
HRS	13.53	12.33	15.35	18.98	19.48	16.51	18.72	18.83	21.73	20.65	18.96	18.73	
PVT without HCC, n												
TIPS	*14*	*29*	*20*	*50*	*58*	*51*	*121*	*130*	*118*	*156*	*160*	*184*	<0.001
*Without TIPS*	*1421*	*1493*	*1494*	*1640*	*1884*	*1984*	*2024*	*2318*	*2429*	*2426*	*2607*	*2819*	<0.001
Complications or comorbidities													
* HE, %*													
* Grade 1*	*27.27*	*32.43*	*36.19*	*29.08*	*29.80*	*28.98*	*27.27*	*34.33*	*35.11*	*40.80*	*38.78*	*38.54*	0.016
* Grade 2*	*18.18*	*16.22*	*27.62*	*21.99*	*17.88*	*18.18*	*26.60*	*25.89*	*27.66*	*24.13*	*23.17*	*19.49*	0.275
* Grade 3*	*14.55*	*22.97*	*11.43*	*14.18*	*17.22*	*15.34*	*14.14*	*14.99*	*16.76*	*15.92*	*14.39*	*17.34*	0.888
* Grade 4*	*3.64*	*6.76*	*4.76*	*7.80*	*6.62*	*4.55*	*9.76*	*4.90*	*5.32*	*2.99*	*5.61*	*4.50*	0.593
*Respiratory disease, %*	*5.57*	*8.55*	*9.37*	*12.59*	*10.33*	*11.41*	*23.19*	*26.36*	*27.07*	*29.06*	*28.04*	*30.88*	<0.001
*Circulatory and neurovascular disease, %*	*25.71*	*35.50*	*35.16*	*40.88*	*40.95*	*42.16*	*79.82*	*82.04*	*80.79*	*79.68*	*81.19*	*83.72*	<0.001
*Heart disease, %*	*1.48*	*2.64*	*1.86*	*2.34*	*2.49*	*2.92*	*6.57*	*6.72*	*6.59*	*6.53*	*7.69*	*7.56*	<0.001
*Obesity, %*	*2.05*	*1.42*	*1.30*	*2.26*	*1.81*	*2.78*	*3.90*	*4.30*	*3.90*	*4.60*	*5.31*	*3.73*	<0.001
*Infections, %*	*6.03*	*7.43*	*8.16*	*10.68*	*10.63*	*10.59*	*21.20*	*21.05*	*22.20*	*22.19*	*24.00*	*23.53*	<0.001
*Diabetes, %*	*0.00*	*0.00*	*0.21*	*0.18*	*0.15*	*0.00*	*0.14*	*0.07*	*0.06*	*0.06*	*0.17*	*0.00*	0.914

Note: *P* values were calculated using linear regression.

Abbreviation: HRS, hepatorenal syndrome.

The median age of patients admitted for TIPS insertion increased significantly from 56 in 2007 to 59 years in 2018 (*p* < 0.001). When stratified according to age groups, the patients aged 50 to 69 years represented the largest population throughout the observation period and covered 58.16% of all TIPS placements, (Figure 1B) Most patients admitted for TIPS insertion were men (67.34%) (Table 1).

Indications for TIPS were classified as gastroesophageal variceal bleeding, recurrent/refractory ascites, and hepatorenal syndrome (HRS). The number of TIPS insertion due to these 3 indications increased significantly during the observation period (*p* < 0.0001). Of all indications, ascites remained the most frequent one with 1364 (57.4%) admissions in 2018, followed by bleedings (544 [20.5%]) and HRS (347 [13.5%]) (Figure 1C). Furthermore, TIPS insertion for ascites increased 7.8 times from 2007 (174) to 2018 (1,364), with an especially steep increase from 2012 (590) to 2013 (990).

When investigating all admissions of cirrhotic patients with bleeding, ascites, or HRS during the entire observation period, only a small percentage (<4%) of cirrhotic patients with these complications received TIPS. However, the proportion of TIPS insertion among cirrhotic patients saw a steep increase during the observation period (*p* < 0.0001). In these patients, those with HRS reached the highest proportion with TIPS insertion of 3.41% in 2018, almost 6 times higher than in 2007 (0.58%). The proportion of TIPS insertion in patients admitted for ascites or bleeding also increased from 0.41% and 0.3% in 2007 to 2.17% and 2.11% in 2018, respectively (Figure. 1D). The number of admissions for TIPS insertion in patients with PVT without HCC experienced a 13-fold increase, from 14 (0.98%) in 2007 to 184 (6.13%) in 2018 (Figure 1E and Supplemental Figure S1A, http://links.lww.com/HC9/A491).

### In-hospital mortality of patients admitted for TIPS according to age, sex, and indications

The in-hospital mortality rate of patients with TIPS insertion increased significantly over time from 4.29% in 2007 to 8.85% in 2018 (*p* < 0.0001) in cirrhosis cases and ranged between 2.94% and 12.89% in non-cirrhotic patients with TIPS placement (Supplemental Figure S1B, http://links.lww.com/HC9/A491). The in-hospital mortality rate was further compared among different age groups (Supplemental Figure S1C, http://links.lww.com/HC9/A491). TIPS placement patients aged between 50 and 69 years had a lower in-hospital mortality rate of 7.25% (50–59 y) and 7.50% (60–69 y compared with patients without TIPS (Supplemental Figure S1C, http://links.lww.com/HC9/A491). However, from 2007 to 2018, there was a slight increase in mortality in TIPS patients from 6.07% to 8.65% in the 50–59 years age group and from 7.02% to 8.17% in the 60–69 years age group. Patients aged between 70 and 79 had a mean mortality rate of 9.92%, whereas older patients (80 and above) had the highest mortality rate of 16.14%, without a statistically significant benefit of survival with TIPS compared to without TIPS (Supplemental Figure S1C and 1D, http://links.lww.com/HC9/A491).

**FIGURE 2:**
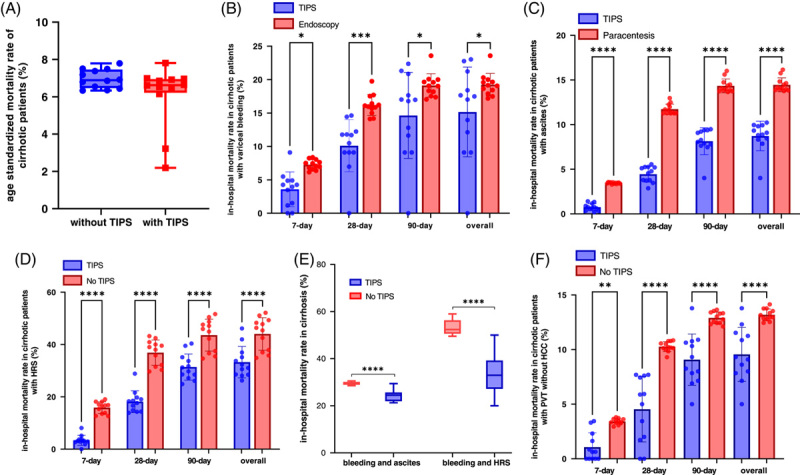
(A) Age-standardized mortality rate among admissions of cirrhotic patients with and without TIPS insertion from 2007 to 2018; no significance was detected with Mann-Whitney *U* test. (B) 7-day, 28-day, 90-day, and overall in-hospital mortality rate among admissions of cirrhotic patients with TIPS insertion and with endoscopy treatment for bleeding without TIPS from 2007 to 2018. (C) 7-day, 28-day, 90-day, and overall in-hospital mortality rate among admissions of cirrhotic patients with TIPS insertion and with paracentesis treatment without TIPS for ascites from 2007 to 2018. (D) 7-day, 28-day, 90-day, and overall in-hospital mortality rate among admissions of cirrhotic patients with and without TIPS insertion for HRS from 2007 to 2018. (E) The in-hospital mortality rate among admissions of patients with bleeding and additional complications of ascites or hepatorenal syndrome in cirrhosis. (F) 7-day, 28-day, 90-day, and overall in-hospital mortality rate among admissions of cirrhotic patients with non-HCC–related PVT, and with or without TIPS. *p < 0.05, **p < 0.01, ***p < 0.001 and ****p < 0.0001 with Mann-Whitney *U* test after multiple test adjustment. Note: The smallest numbers that have been anonymized due to data protection were replaced with 1 in the analysis. Abbreviation: HRS, hepatorenal syndrome.

To eliminate the impact of age on in-hospital mortality, the age-standardized mortality rate was calculated. The age-standardized mortality rate of cirrhotic patients without TIPS insertion ranged between 7.79% in 2007 and 6.54% in 2018. The age-standardized mortality rate of TIPS patients with cirrhosis increased from 2007 (2.2%) to 2009 (6.4%), while from 2009 to 2018, it ranged between 6.2% and 7.8%, similar to the age-standardized mortality rate of liver cirrhosis patients. The mortality rates of patients with (6.11%) and without TIPS (6.96%) were similar (Figure 2A). as were the mortality rates between male (8.64%) and female (7.27%) patients.

Next, in-hospital mortality was compared between the different indications for TIPS (bleeding, ascites, and HRS) (Figure 2C). No significant difference could be detected in the mortality rate when comparing admissions of patients with bleeding receiving TIPS and patients not receiving TIPS (Supplemental Figure S2, http://links.lww.com/HC9/A491). Interestingly, patients admitted for bleeding and receiving TIPS had a significantly lower in-hospital mortality rate compared with patients treated only with endoscopy (overall: 15.2% vs. 19.2%, *p* = 0.002) (Figure 2B). Similarly, the mortality rate in patients with TIPS insertion for ascites and HRS was significantly lower than in those without TIPS or with paracentesis (ascites: 8.7% vs. 14.4%, *p* < 0.0001; HRS: 17.1% vs. 43.3%, *p* < 0.0001) (Figures 2C and 2D, Supplemental Figure S2, http://links.lww.com/HC9/A491). In cirrhotic patients with bleeding and additional ascites or HRS, TIPS placement resulted in a more noticeable reduction of mortality rate, especially in patients with bleeding and HRS (53.5% vs. 33.3%) (Figure 2E). Correspondingly, we also compared the outcome of patients admitted with coagulation failure treated with or without TIPS and found a significant improvement in survival in the TIPS group (Supplemental Figures S3A and B, http://links.lww.com/HC9/A491).

In addition, we separated TIPS patients according to the type of stent, if coded, namely covered or uncovered TIPS. The vast majority of patients received covered TIPS (76%) for ascites, and the number doubled from 527 in 2013 to 1026 in 2018. Remarkably, the mortality rate of ascites patients with covered TIPS was lower compared with patients who received an uncovered TIPS, especially in the long-term (covered vs. uncovered 90 d: 5.0% vs. 8.2%, *p* < 0.0001; overall: 9.7% vs. 11.1%, *p* = 0.001) (Supplemental Figure S3C, http://links.lww.com/HC9/A491).

Since these data might be biased by the severity of liver dysfunction, the impact of TIPS insertion on the mortality rate of cirrhotic patients with hypoalbuminemia or hyperbilirubinemia diagnosis was investigated. While TIPS reduced the mortality rate in both patient groups, this was only statistically significant for hypoalbuminemia, with a decrease from 21.10% to 16.10% in patients with hypoalbuminemia (*p* = 0.027) and from 19.75% to 15.81% in patients with hyperbilirubinemia compared with non-TIPS, which was not statistically significant (*p* = 0.659) (Supplemental Figure S3D, http://links.lww.com/HC9/A491). Therefore, we further performed subgroup analysis in patients admitted with ascites or HRS with additional hyperbilirubinemia. In both groups, TIPS insertion showed a significant improvement of the survival despite hyperbilirubinemia (mortality rate ascites: 12.4% vs. 23.6%, *p* = 0.03; HRS: 2.8% vs. 52.6%, *p* < 0.0001) (Supplemental Figure S2E, http://links.lww.com/HC9/A491).

The mortality rate in cirrhotic patients admitted with PVT and TIPS insertion (10.18%) decreased during the observation period from 13.3% in 2017 to 10.7% in 2018 (Supplemental Figure S2F, http://links.lww.com/HC9/A491). The 7-day, 28-day, and 90-day mortality rates were all lower in patients with PVT without HCC admitted for TIPS insertion compared with that found in patients without TIPS (overall mortality rate: 9.6% vs. 13.3%, *p* < 0.0001) (Figure 2F).

### Hepatic encephalopathy and other comorbidities

Since post-shunt HE frequently occurs after TIPS placement, the impact of HE was investigated. During the observed period, the percentage of in-hospital diagnoses of different grades of HE development after TIPS insertion remained stable (Supplemental Figures S4A and B, http://links.lww.com/HC9/A491), despite an increase in the total number of HE diagnoses from 18.15% in 2007 to 25.20% in 2018 (Supplemental Figure S4C, http://links.lww.com/HC9/A491).

**FIGURE 3:**
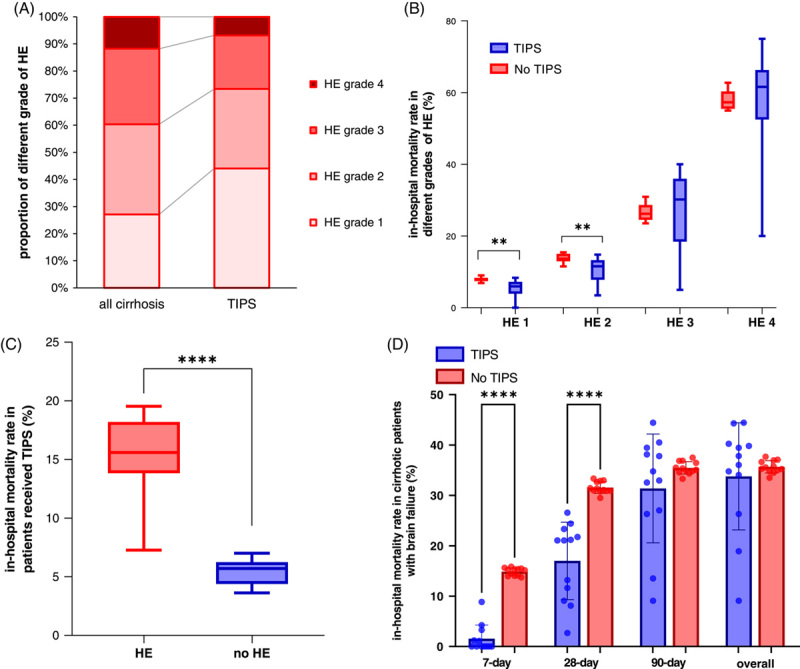
(A) Proportion of different grades of HE in all admissions of patients with cirrhosis and patients admitted for TIPS insertion from 2007 to 2018. (B) The in-hospital mortality rate among admissions in patients with different grades of HE with or without TIPS insertions from 2007 to 2018. (C) The in-hospital mortality rate among admissions of cirrhotic patients admitted for TIPS insertions with and without HE from 2007 to 2018. (D) 7-day, 28-day, 90-day, and overall in-hospital mortality rate among admissions of patients with brain failure, with or without TIPS. **p < 0.01, ****p < 0.0001 with Mann-Whitney *U* test after multiple test adjustments. Note: The smallest numbers that have been anonymized due to data protection were replaced with 1 in the analysis.

By contrast, in the cirrhotic patients admitted without TIPS placement, grade 1 HE increased and grade 4 HE decreased in 2018, while HE grades 2 and 3 remained stable (Supplemental Figure S3B, http://links.lww.com/HC9/A491). The percentage of patients with lower grades of HE (grades 1 and 2) account for the majority, both in patients with and without TIPS (Figure 3A). Surprisingly, the proportion of admissions with HE grade 1 in cirrhotic patients with TIPS (44.1%) was almost twice as high as the one found in patients without TIPS placement (27.1%), with 50% in 2018 (Figure 3A).

As expected, cirrhotic patients with HE grade 4 with or without TIPS placement had the highest mortality rate (58.5% and 57.9%), while patients with HE grade 1 had the lowest mortality rate (5.4% in TIPS patients and 7.9% in patients without TIPS). Importantly, TIPS patients with HE grade 1 or grade 2 had significantly lower mortality rates (5.4% and 10.4%) compared with the non-TIPS (7.9% and 13.8%) (Figure 3B). However, the in-hospital mortality rate in cirrhotic patients with TIPS was almost 4 times higher in HE patients than in patients without HE (19.06% vs. 5.41%, *p* < 0.0001) (Figure 3C). Furthermore, the short-term mortality rate (TIPS vs. non-TIPS 7-day: 1.6% vs.14.9%, *p* < 0.0001, and 28-day: 17.0% vs. 31.6%, *p* < 0.0001) showed a significant decrease among admissions of patients with brain failure (HE grades 3 or 4) receiving TIPS compared with those without TIPS (Figure 3D). Similar trends could be observed in admissions of patients for ascites with additional diagnosis of HE (Supplemental Figure S4D, http://links.lww.com/HC9/A491).

Possible comorbidities of cirrhotic patients with TIPS were categorized into 6 groups. Of these, circulatory diseases account for the vast majority (58.04%), followed by respiratory disease (18.5%) and infections (15.6%) (Table 1 and Supplemental Figure S4E, http://links.lww.com/HC9/A491). Nevertheless, the number of these 6 comorbidities increased roughly 3-fold during the observation period, especially regarding circulatory diseases (Table 1 and Supplemental Figure S4E, http://links.lww.com/HC9/A491). Interestingly, the number of admissions for TIPS in cirrhotic patients with respiratory diseases had the highest mortality rate (26.7%), exceeding infections (22.7%). Survival benefit due to TIPS insertion could only be found in patients with circulatory diseases (8.6% vs. 10.4%, *p* < 0.0001) or infections (18.2% vs. 20.5%, *p* < 0.01) as comorbidities of cirrhosis (Supplemental Figure S4F, http://links.lww.com/HC9/A491).

### Logistic regression of in-hospital mortality rate

**FIGURE 4:**
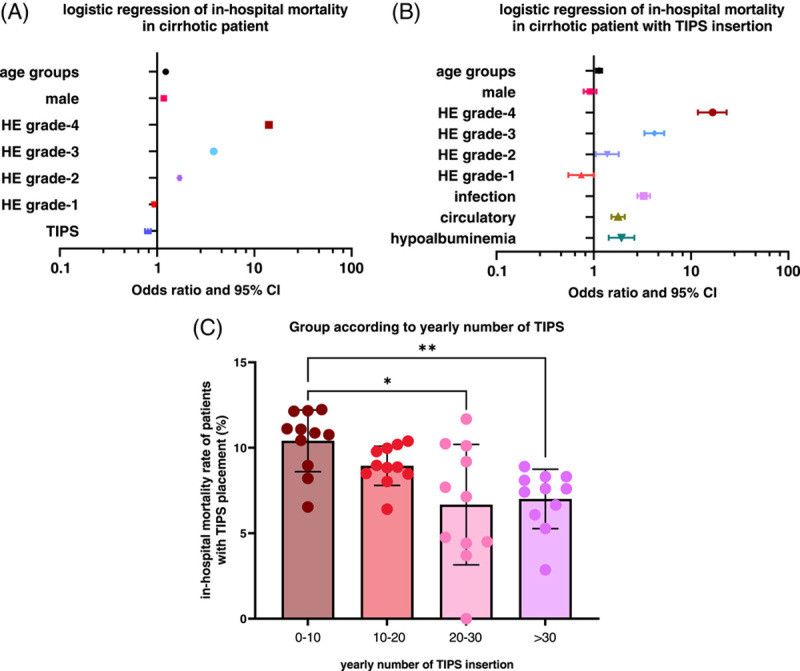
(A) Forest plot of significant risk factors of in-hospital mortality among admissions of cirrhotic patients from 2007 to 2018 using a multivariable logistic regression model. (B) Forest plot of risk factors of in-hospital mortality among admissions of cirrhotic patients with TIPS insertion from 2007 to 2018 using a multivariable logistic regression model. Models were adjusted by age groups, sex, different grades of HE, infection, circulatory diseases, hypoalbuminemia, and hyperbilirubinemia. Male sex and hyperbilirubinemia were not statistically significant. Note: Ages were grouped into 5 groups: < 50, 50–59, 60–69, 70–79, and > 80 years old. Different grades of HE were compared with no-HE. (C) The in-hospital mortality rate among admissions of patients for TIPS insertions. Comparisons were made between different groups according to the yearly number of TIPS insertions in each TIPS center from 2007 to 2018. **p < 0.01, and *p < 0.05 with Mann-Whitney *U* test after multiple test adjustments.

Multivariable logistic regression models were performed to determine the impact of TIPS insertion and other potential independent risk factors on the in-hospital mortality rate. As shown in Figure 4A and Table 2, TIPS insertion (odds ratio [OR]: 0.81 [95% CI: 0.75–0.86]) is independently associated with a lower risk of in-hospital mortality, despite the fact that the OR of HE increased in parallel with grades of in-hospital mortality. HE grade 1 was not shown to be associated with a high risk of in-hospital mortality (OR: 0.93 [95% CI: 0.90–0.96]). Similar results can be observed when admissions of patients were separated into different groups according to the length of hospital stay (7-day, 28-day, and 90-day) (Supplemental Figures S5A, B and C, http://links.lww.com/HC9/A491).

**TABLE 2:** Logistic regression of potential risk factors on in-hospital mortality in patients with cirrhotic and in the subgroup of cirrhotic patients admitted for TIPS insertion from 2007 to 2018

Variable	OR and 95% CI	*p*
*Cirrhosis (n = 1,823,845)*		
Age groups	1.23 (*1.22*–1.23)	<.0001
Male	1.17 (1.16–1.18)	<.0001
HE grade 4	14.49 (*13.70–14.49*)	<.0001
HE grade 3	*3.82* (*3.73–3.89*)	<.0001
HE grade 2	*1.70* (*1.66–1.75*)	<.0001
HE grade 1	0.9*3* (0.9*0–0.96*)	<.0001
TIPS	0.8*1* (0.7*5–0.86*)	<.0001
*TIPS (n = 11,312)*		
Age groups	*1.14* (*1.06–1.22)*	0.002
Male	*0.92* (*0.79–1.07)*	0.266
HE grade 4	*16.67* (*11.76–23.26)*	<.0001
HE grade 3	*4.20* (*3.32–5.29)*	<.0001
HE grade 2	*1.38* (*1.05–1.81)*	<.0001
HE grade 1	*0.75* (*0.55–1.00)*	<.0001
Infection	*3.27* (*2.82–3.79)*	<.0001
Circulatory diseases	*1.78* (*1.43–3.42)*	<.0001
Hypoalbuminemia	*1.93* (*1.43–2.62)*	<.0001
Hyperbilirubinemia	*1.66* (*0.80–3.42)*	0.172

*Note:* Ages were grouped into 5 groups: < 50, 50–59, 60–69, 70–79, and >80 years old. Different grades of HE were compared with no-HE.

Furthermore, we performed multivariable logistic regression of in-hospital mortality among patients admitted for TIPS insertions adjusted for all potential risk factors, including complications and comorbidities. Of note, lower HE grades (grades 1 and 2) had less impact on in-hospital mortality than higher HE grades (grades 3 and 4). Especially HE grade 1 was shown not to be an independent risk factor for in-hospital mortality (OR: 0.75 [95% CI: 0.55–1.00]) (Figure 4B and Table 2). While hypoalbuminemia was not found to be an independent risk factor for mortality among admissions of patients with cirrhosis, it nevertheless increased the risk of mortality in patients after TIPS insertion (Figure 4B), especially in the long-term (90-day and overall mortality) (Supplemental Figures S5D, E and F, http://links.lww.com/HC9/A491). In line with previous results, infections (OR: 3.27 [95% CI: 2.82–3.79]) and circulatory diseases (OR: 1.78 [95% CI: 1.43–3.42]) were the only significant, independent risk factors for in-hospital mortality of the other comorbidities listed in our analysis (Figure 4B). Findings were similar in the different subgroups of admission of patients with different length of hospital stay (Supplemental Figures S5D, E and F, http://links.lww.com/HC9/A491) and for different indications, such as bleeding, ascites and HRS (Table 3).

**TABLE 3:** Logistic regression of potential risk factors on in-hospital mortality in the subgroup of cirrhotic patients admitted with bleeding, ascites, or HRS

		95% CI		
Liver cirrhosis	OR	Lower	Upper	*p*
Bleeding				
Age	1.011	1.010	1.012	<0.0001
Male	1.100	1.080	1.121	<0.0001
TIPS insertion	0.838	0.772	0.909	<0.0001
HE	2.513	2.463	2.571	<0.0001
Ascites				
Age	1.017	1.017	1.018	<0.0001
Male	1.050	1.035	1.066	<0.0001
TIPS insertion	0.570	0.531	0.612	<0.0001
HE	2.421	2.387	2.463	<0.0001
HRS				
Age	1.015	1.014	1.016	<0.0001
Male	1.019	0.993	1.047	0.1508
TIPS insertion	0.298	0.268	0.331	<0.0001
HE	1.357	1.323	1.393	<0.0001

Abbreviation: HRS, hepatorenal syndrome.

### Training effect

To evaluate the training effect, we classified the admissions of patients from different TIPS centers according to the yearly number of TIPS insertions. We found that the in-hospital mortality rate was associated with the proficiency of the TIPS procedure in the hospital where it was performed. That is, the number of TIPS procedures is inversely proportional to the mortality rate. The in-hospital mortality rate of the patients with TIPS insertion from the centers with less than 10 TIPS insertions (10.4%) per year was significantly higher than those with 20 to 30 TIPS insertions per year (6.7%, *p* = 0.044), and highly significant compared with centers with 30 or more TIPS insertions (7.0%, *p* = 0.007) (Figure 4C).

## DISCUSSION

The present nationwide study analyzed almost 15,000 TIPS insertions over a period of 12 years in Germany. During this time period, TIPS was increasingly used for portal hypertensive complications. In earlier years, only a few carefully selected patients had access to TIPS, and only low-risk TIPS insertions were performed, accompanied by a rather low mortality rate over time. Wider use of TIPS in more severe patients led to an increase of in-hospital mortality, similar to overall in-hospital mortality in cirrhosis patients as described elsewhere.^24^ Patients benefited from the treatment with TIPS, regardless of indication, with a better survival rate. In addition, TIPS procedures seem to be safe in patients with lower HE grades.

Insertion of TIPS stents was increasingly observed in the 12 observed years in Germany. Especially in the years 2011 to 2013, after the publication of the Baveno V consensus report and the subsequent recommendation in German guidelines for preemptive TIPS for gastroesophageal variceal bleeding and TIPS implementation as first-line treatment in patients with refractory or recurrent ascites,^25^ an almost 4-fold increase in TIPS utilization was observed.^26^ The number of TIPS insertions for different indications increased in parallel with the number of respective complications of liver cirrhosis.^24^ Especially in patients with ascites, who still account for the vast majority of patients with decompensation of cirrhosis, a steep increase in the number of admissions as well as TIPS insertions has occurred.^24^ Interestingly, the number of patients undergoing TIPS for bleeding also showed an increase, and this may be 1 reason for the overall decrease in numbers of variceal bleeding.^18^ Similarly, TIPS revision was performed in around 10% of all TIPS treatments, which shows another increase.

Our data show that TIPS insertion and revision improved the survival of patients independent of different indications and most relevantly in patients with cirrhosis admitted for TIPS for ascites or HRS. While the in-hospital mortality rate of patients admitted for TIPS insertion increased slightly between 2007 and 2009, it has since remained at a steady level, as has overall cirrhosis in-hospital mortality. It should be highlighted that more severely ill patients were increasingly included in the procedures in the later era of the observation period due to expanding inclusion criteria, while in the early observation period, selection concentrated on low-risk patients who benefitted from survival following TIPS insertion, especially for variceal bleeding, without high risk.^19,27^

Contrary to the general belief that hypoalbuminemia and hyperbilirubinemia are associated with a high risk of death and complications,^28^ patients with hypoalbuminemia had a significantly better outcome after TIPS insertion. In addition, patients with higher bilirubin levels did not show an increase in the risk of death after TIPS, but there was a large discrepancy between patients. However, due to the limitation of the data, we could not differentiate liver failure (bilirubin over 12 mg/dl) in these patients. Yet, hyperbilirubinemia may not be an absolute contraindication for TIPS, for example, in patients with variceal bleeding. Careful evaluation of benefits and risks should be done for each patient, as shown in our recent publication on TIPS patients with ACLF.^18^ While our data reflect the reality of admissions of patients selected for TIPS insertion, the futility criteria of TIPS insertion need further investigations to compare the outcome of patients with or without TIPS.

Surprisingly, the vast majority of TIPS patients were diagnosed with lower-grade HE (grades 1 or 2). Patients with lower HE grades seem to benefit from TIPS implantation and had significantly better in-hospital survival after TIPS insertion compared with those without it. This was also shown by several studies,^21,29–33^ namely that pre-TIPS, HE is not predictive of survival and not a contraindication for TIPS. Only severe or uncontrolled HE without clear precipitants might be taken into consideration as a relative contraindication. Especially for refractory ascites, the pros of survival benefits outweigh the cons of developing post-TIPS HE. This may also be due to the use of rifaximin as prophylaxis to prevent overt HE and the application of covered TIPS with smaller diameters.^21,34^

Our study has several limitations. First, admission numbers instead of patients were used for analysis due to data protection regulations. Therefore, follow-up of a single patient was not available, and patients with multiple admissions may be over-estimated, leading to a bias in the analysis. For the same reason, data as to whether HE developed before or after TIPS insertion was also not available. Second, the cause of in-hospital mortality could not be retrieved. However, the nationwide epidemiological data of all admissions for TIPS insertion does provide strong evidence for the management of the patients. Another limitation is that patients with HRS could not be differentiated according to the type of existing kidney injury. Furthermore, given that the German DRG system has met considerable criticism with regard to the complicated coding since its establishment,^35^ the inaccuracy of coding due to manual reporting by the responsible physician cannot be excluded. However, as the reimbursement system is based on the DRG system, the coding, especially for interventions, is important and must be correct.

In conclusion, the present study of nationwide epidemiological data of TIPS placement shows promising trends in Germany for the observed 12 years, with a steeply increased number of patients benefitting from TIPS insertion regardless of indications. TIPS procedures appear to be safe in patients with lower grades of HE, hypoalbuminemia, and/or hyperbilirubinemia. Further large-scale prospective studies are needed to analyze the futility criteria for TIPS insertion.

## Supplementary Material
