# Supplementary material for: Use and outcome of TIPS in hospitalized patients in Germany: A Nationwide study (2007–2018)
Source: Hepatol Commun. 2023 Sep 15;7(10):e0237. doi: 10.1097/HC9.0000000000000237 (PMC10503680; doi:10.1097/HC9.0000000000000237)
Supplement: Supplementary file 1 [file hc9-7-e0237-s001.docx]

**Supplementary material**

**Content**

| **Table or Figure** | **Content** | **Page** |
| --- | --- | --- |
| Supplementary Table 1 | ICD or OPS code of the diagnosis or procedures | 2 |
| Supplementary Figure 1 | In-hospital death rate among admissions of patients with TIPS placement in different year groups, of patients admitted with cirrhosis and admitted for TIPS in each age group, of patients admitted for TIPS placement in different year and age groups, and of patients with bleeding, ascites or hepatorenal syndrome in cirrhosis with or without TIPS insertion. Number of admissions of patients with portal vein thrombosis including hepatocellular carcinoma with or without TIPS insertion and proportion of TIPS insertions among all admissions of patients with PVT | 6 |
| Supplementary Figure 2 | In-hospital death rate among admissions of patients with bleeding, ascites or hepatorenal syndrome in cirrhosis with or without TIPS insertion, of patients with endoscopy with or without TIPS insertion in the same hospital stay for bleeding, of patients with TIPS insertion or paracentesis for ascites and of patients with or without TIPS for HRS from 2013 to 2018 separately by year | 7 |
| Supplementary Figure 3 | 7-day, 28-day, 90-day and overall in-hospital death rate among admissions of patients with or without TIPS insertion for coagulation failure and separated by year, of patients with covered or uncovered TIPS for ascites, of cirrhotic patients with hypalbuminemia or hyperbilirubinemia, with or without TIPS insertion, of cirrhotic patients for ascites or HRS with hyperbilirubinemia, with or without TIPS insertion and of cirrhotic patients with PVT, and with or without TIPS | 8 |
| Supplementary Figure 4 | Proportion of different grades of HE in admissions of cirrhotic patients who received TIPS, and in all admissions of patients with HE. Proportion of in-hospital HE in admissions of patients with cirrhosis for TIPS placement. 7-day, 28-day, 90-day and overall in-hospital death rate among admissions of cirrhotic patients with ascites and HE. Number of deaths of admissions from cirrhotic patients with TIPS with different complications. In-hospital death rate among admissions of different comorbidities in cirrhotic patients with or without TIPS insertion | 9 |
| Supplementary Figure 5 | Logistic regression model of in-hospital mortality for different times of hospital stay | 10 |
| Supplementary Figure 5 | Number and logistic regression in patients with TIPS revision | 11 |

**Supplementary Table 1.** ICD or OPS code of the diagnosis or procedures

| **Variables** | **OPS** | **Definitions** |
| --- | --- | --- |
| TIPS insertion | 8-839.81, 8-839.82, 8-839.83, 8-839.85, 8-839.87, 8-839.88, 8-839.89, 8-839.8a, 8-839.8x | Portosystemic shunt including percutaneous (balloon) angioplasty, percutaneous thrombolysis, percutaneous thrombectomy, percutaneous reduction of an existing portosystemic shunt, percutaneous closure, percutaneous placement of an uncovered stent, percutaneous placement of a covered stent, percutaneous placement of 2 or more uncovered stents, percutaneous placement of 2 or more covered stents and other |
| Covered TIPS insertion | 8-839.88, 8-839.8a | Percutaneous placement of a covered stent, and percutaneous placement of 2 or more covered stents |
| Uncovered TIPS insertion | 8-839.87, 8-839.89 | Percutaneous placement of an uncovered stent, and percutaneous placement of 2 or more uncovered stents |
| TIPS revision | 8-839.86, 8-839.84 | Portosystemic shunt including revision with stent insertion and percutaneous reduction of an existing portosystemic shunt |
| Esophagogastroduodenoscopy treatment | 5-449.03, 5-449.13, 5-449.23, 5-449.83, 5-449.d3, 5-449.e3, 5-449.h3, 5-449.j3, 5-449.k3, 5-449.t3, 5-449.v3, 5-449.s3 | Endoscopic sclerotherapy of fundus varices, endoscopic stenosis of fundus varices, endoscopic locking operation on the fundus, endoscopic ligation (banding) of fundus varices, endoscopic clipping, endoscopic injection, endoscopic insertion or change of a self-expanding prosthesis, endoscopic removal of a self-expanding prosthesis, endoscopic insertion or change of a non-self-expanding prosthesis, endoscopic haemostasis by application of absorbent substances, endoscopic haemostasis by application of peptide hydrogel-forming substances, endoscopic tissue creation or closure by a ring-shaped clip placed on an endoscope |
| Paracentesis | 8-153 | Therapeutic percutaneous puncture of the abdominal cavity. (This code is to be reported only once per inpatient stay)  Excluding Irrigation of the abdominal cavity,  Diagnostic ascites puncture, therapeutic drainage of peritoneal space and therapeutic pelvic drainage. |
| Liver transplantation | 5-504 | Liver transplantation, excluding allogeneic hepatocyte transplantation |
|  | **ICD - 10** |  |
| Liver cirrhosis | K74, K70.3 | Fibrosis and cirrhosis of the liver and alcoholic cirrhosis of the liver |
| Bleeding | D62, K92.0, I98.3, I85.0 | Acute bleeding anemia, hematemesis, esophageal and gastric varices in diseases classified elsewhere, with an indication of bleeding including esophageal and gastric varices with indication of bleeding in liver diseases, esophageal and gastric varices with indication of bleeding in schistosomiasis and esophageal varices with bleeding |
| Variceal bleeding | I98.3 | Oesophageal and gastric varices in diseases classified elsewhere, with indication of haemorrhage. Including oesophageal and gastric varices with indication of haemorrhage in liver diseases, oesophageal and gastric varices with indication of haemorrhage in schistosomiasis |
| Ascites | R18 | Ascites including accumulation of fluid in the abdominal cavity |
| Hepatorenal syndrome | K76.7 | Hepatorenal syndrome |
| Kidney failure | K76.7, N17, 8-85a | Hepatorenal syndrome, acute renal failure  Including acute kidney injury [AKI], acute renal failure, acute kidney injury and acute prerenal renal failure.  Dialysis procedure due to lack of function and failure of a kidney transplant. |
| Hepatic encephalopathy | K72.7  Grade 1: K72.71  Grade 2: K72.72  Grade 3: K72.73  Grade 4: K72.74 | Hepatic encephalopathy and coma hepaticum: grading is based on West Haven criteria.  Grade 1 hepatic encephalopathy: euphoria or anxiety; attention deficit; difficulty adding up.  Grade 2 hepatic encephalopathy: lethargy, apathy; minimal disorientation to time and space; subtle personality changes; inappropriate behavior; difficulty subtracting.  Grade 3 hepatic encephalopathy: somnolence to stupor; response to verbal stimuli; confusion; disorientation to time and space.  Grade 4 hepatic encephalopathy: coma; no response to verbal stimuli or pain stimulation |
| Brain failure | Grade 3: K72.73  Grade 4: K72.74 | Grade 3 hepatic encephalopathy: somnolence to stupor; response to verbal stimuli; confusion; disorientation to time and space.  Grade 4 hepatic encephalopathy: coma; no response to verbal stimuli or pain stimulation |
| Liver failure | 8-85 and R17 | Hyperbilirubinemia, with or without jaundice, not elsewhere classified and extracorporeal liver replacement therapy [liver dialysis]. |
| Coagulation failure | D68.4 | Acquired deficiency of clotting factors, including coagulation factor deficiency due to liver disease and coagulation factor deficiency due to vitamin K deficiency |
| Hyperbilirubinemia | R17 | Hyperbilirubinemia, with or without jaundice, not elsewhere classified |
| Hypoalbuminemia | R77.0 | Changes in albumins |
| Portal vein thrombosis | I81 | portal vein thrombosis incl. portal vein occlusion; excluding portal vein phlebitis |
| Hepatocellular carcinoma | C22 | Malignant neoplasm of the liver and intrahepatic bile ducts |
| Heart diseases | I20, I21, I22, I23, I24, I25 | Angina pectoris, acute myocardial infarction, recurrent myocardial infarction, certain acute complications after acute myocardial infarction, other acute ischemic heart disease and chronic ischemic heart disease |
| Circulatory diseases | I00-I99, excluding heart diseases | Acute rheumatic fever, chronic rheumatic heart diseases, pulmonary heart disease and diseases of the pulmonary circulation, other forms of heart disease, cerebrovascular diseases, diseases of the arteries, arterioles and capillaries, diseases of the veins, lymphatic vessels, and lymph nodes, not elsewhere classified, other and unspecified diseases of the circulatory system |
| Respiratory diseases | J00 – J99, excluding J00-J18 and J20-22 | Other acute lower respiratory infections, other upper respiratory diseases, chronic lower respiratory diseases, lung diseases caused by exogenous substances, other respiratory diseases mainly affecting the interstitium, purulent and necrotizing lower respiratory diseases, other diseases of the pleura and other diseases of the respiratory system |
| Infections | J00, J01, J02, J03, J04, J05, J06, J07, J08, J09, J10, J11, J12, J13, J14, J15, J16, J17, J18, J20, J21, J22, L01, L02, L03, M72, K65, N390 | Pneumonia; urinary tract infection; skin and subcutaneous infections; fibromatoses and peritonitis |

**Supplementary Figure 1**

**A B**

**C D**

**Supplementary Figure 1. A.** Number of admissions of patients with portal vein thrombosis including hepatocellular carcinoma with or without TIPS insertion and proportion of TIPS insertions among all admissions of patients with PVT. ****, p < 0.0001 with linear regression **B.** In-hospital death rate among admissions of patients with TIPS placement in different year groups **C.** In-hospital death rate among hospital admissions of patients admitted with cirrhosis and admitted for TIPS in each age group from 2007 to 2018 **D.** In-hospital death rate among admissions of patients admitted for TIPS placement in different year and age groups

Abbreviations: PVT, portal vein thrombosis

**Supplementary Figure 2**

**A B**

**C**

**D**

**Supplementary Figure 2. A.** In-hospital death rate among admissions of patients with bleeding, ascites or hepatorenal syndrome in cirrhosis with or without TIPS insertion. ****, p < 0.0001, ns, not significant with Mann-Whitney U test **B**. 7-day, 28-day, 90-day and overall in-hospital death rate among admissions of patients with endoscopy with or without TIPS insertion during the same hospital stay for bleeding from 2013 to 2018 **C.** 7-day, 28-day, 90-day and overall in-hospital death rate among admissions of patients with TIPS insertion or paracentesis for ascites from 2013 to 2018 separated by year **D.** 7-day, 28-day, 90-day and overall in-hospital death rate among admissions of patients with or without TIPS for HRS from 2013 to 2018 separate by year

Note: The smallest numbers that have been anonymized due to data protection were replaced with 1 in the analysis. Abbreviations: TIPS; transjugular intrahepatic portosystemic shunts; HRS, hepatorenal syndrome

**Supplementary Figure 3**

**A B**

**C D**

**E F**

**Supplementary Figure 3. A.** 7-day, 28-day, 90-day and overall in-hospital death rate among admissions of patients with or without TIPS insertion for coagulation failure from 2007 to 2018, ****, p < 0.0001 with Mann-Whitney U test after multiple test adjustment **B.** separated by year **C.** In-hospital death rate among admissions of patients with covered or uncovered TIPS for ascites, *, p < 0.05 with Mann-Whitney U test, **, p < 0.01 **D**. In-hospital death rate among admissions of cirrhotic patients with hypalbuminemia or hyperbilirubinemia, with or without TIPS insertion, *, p < 0.05, with Mann-Whitney U test after multiple test adjustment **E.** In-hospital death rate among admissions of cirrhotic patients for ascites or HRS with hyperbilirubinemia, with or without TIPS insertion *, p < 0.05, ****, p < 0.0001 with Mann-Whitney U test after multiple test adjustment **F**. In-hospital death rate among admissions of cirrhotic patients with PVT, and with or without TIPS, separated by year from 2007 to 2018, *, p < 0.05 with linear regression. Abbreviations: TIPS; transjugular intrahepatic portosystemic shunts; HRS, hepatorenal syndrome

**Supplementary Figure 4**

**A B**

**
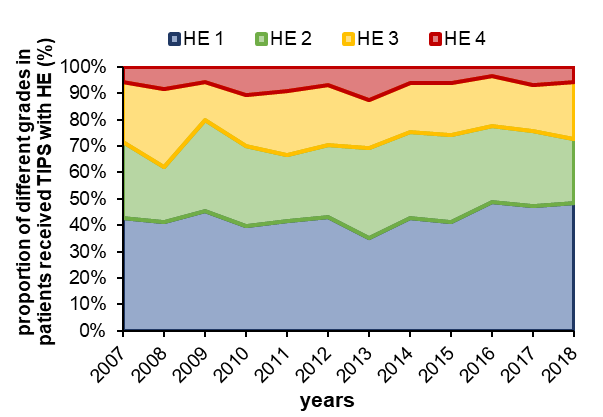

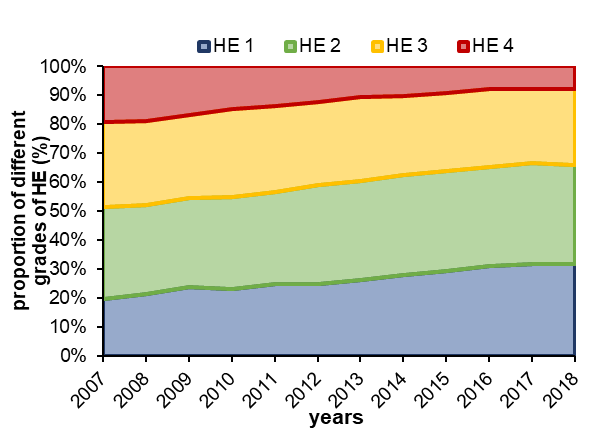
**

**C D**

**E F**

**
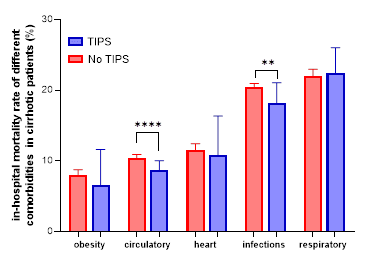
**

**Supplementary Figure 4. A.** Proportion of different grades of HE in admissions of cirrhotic patients who received TIPS from 2007 to 2018 **B.** Proportion of different grades of HE in all admissions of patients with HE from 2007 to 2018 **C.** Proportion of in-hospital HE in admissions of patients with cirrhosis for TIPS placement from 2007 to 2018 **D.** 7-day, 28-day, 90-day and overall in-hospital death rate among admissions of cirrhotic patients with ascites and HE from 2007 to 2018, *, p < 0.05, ***, p < 0.01, and ****, p < 0.0001 with Mann-Whitney U test after multiple test adjustment **E**. Number of deaths of admissions from cirrhotic patients with TIPS with different complications from 2007 to 2018, ****, p < 0.0001 with linear regression **F.** In-hospital death rate among admissions of different comorbidities in cirrhotic patients with or without TIPS insertion, ****, p < 0.0001, **, p < 0.01 with Mann-Whitney U test. Abbreviations: TIPS; transjugular intrahepatic portosystemic shunts; HE, hepatic encephalopathy

**Supplementary Figure 5**

**A B**

**C D**

**E F**

**Supplementary Figure 5.** Forrest plot of OR from multivariable logistic regression of in-hospital mortality in patients with cirrhosis **A.** 7-day; **B**.28-day; **C**. 90-day, adjusted by age groups, sex, different grade of HE and TIPS insertion **D**. 7-day TIPS patients, 28-day TIPS patients **F**. 90-day TIPS patients, model was adjusted by age groups, sex, different grade of HE, infection, circulatory diseases, hypoalbuminemia and hyperbilirubinemia. Male sex was not statistically significant. Age was grouped into five groups: < 50, 50-59, 60-69, 70-79 and > 80 years old. Abbreviations: CI: confidence interval; HE, hepatic encephalopathy; TIPS, transjugular intrahepatic portosystemic shunts; OR, odds ratio

**Supplementary Figure 6**

**A B**

**
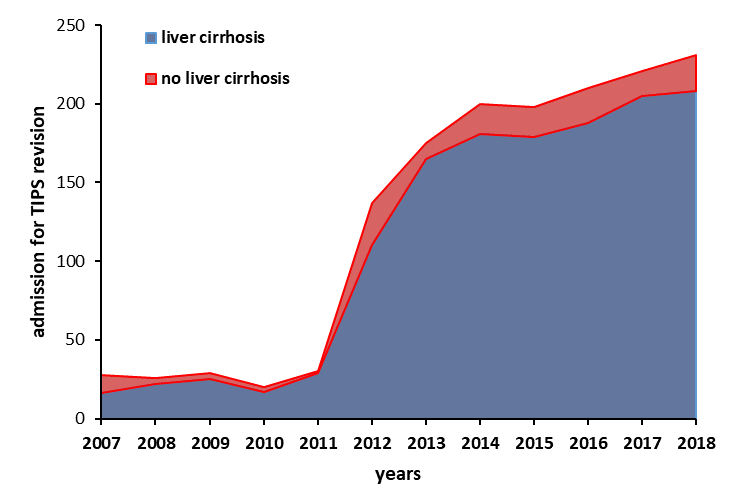
**

**C**.

**Supplementary Figure 6.** **A.** Numbers of admissions with TIPS revision with and without liver cirrhosis from 2007 to 2018 **B.** Forest plot of OR from logistic regression of in-hospital mortality in admissions of cirrhotic patients **C.** Forest plot of OR from logistic regression of in-hospital mortality in admissions of cirrhotic patients admitted for TIPS revision

Abbreviations: CI, confidence interval; HE, hepatic encephalopathy; TIPS, transjugular intrahepatic portosystemic shunts; OR, odds ratio
